# Appropriate Distraction Strength for Metatarsophalangeal Joint Arthroscopy

**DOI:** 10.3390/medicina61040654

**Published:** 2025-04-02

**Authors:** Jong-Kil Kim, Kwang-Bok Lee, Do-Yeon Kim

**Affiliations:** 1 Department of Orthopaedic Surgery, Presbyterian Medical Center, Jeonju 54987, Republic of Korea; andromeda0115@hanmail.net; 2 Department of Orthopaedic Surgery, Research Institute of Clinical Medicine of Jeonbuk National University, Biomedical Research Institute of Jeonbuk National University Hospital, Jeonbuk National University Medical School, Jeonju 54907, Republic of Korea; 3 Saeyeon Orthopaedic Clinic, Jeonju 54907, Republic of Korea; sakdy@hanmail.net

**Keywords:** MTP joint arthroscopy, foot ankle surgery, distraction

## Abstract

*Background and Objectives*: To investigate the natural metatasophalangeal (MTP) joint distance, we studied the appropriate degree of distraction for arthroscopy and the associated factors, including age, gender, and body mass index (BMI). *Materials and Methods*: Sixty-seven patients who underwent MTP joint arthroscopy or foot and ankle surgery from April 2013 to June 2020 were enrolled. Foot plain radiographs were taken using a mini-fluoroscan with no traction, manual traction, and traction of 5 pounds, 10 pounds, and 15 pounds to measure the MTP joint distance. Age, gender, and BMI were compared as associated factors. The minimum joint distance of MTP joint arthroscopy was defined as 2.8 mm, which was the sheath size of a 1.9 mm, 30° high-definition arthroscope. *Results*: Regarding natural MTP joint space sizes, the MTP-2 joint had the largest joint size (2.39 ± 0.37 mm). The MTP-5 joint had the smallest joint size (1.59 ± 0.34 mm). Traction of 10 lb was an appropriate distraction force for the MTP-1 joint (3.09 ± 0.03 mm) and MTP-4 joint (3.07± 0.47 mm) in arthroscopy. Traction of 5lb was an appropriate distraction force for the MTP-2 (3.32 ± 0.60 mm), MTP-3 (2.89 ± 0.50 mm), and MTP-5 (2.97 ± 0.49 mm) joints. For the MTP-1 and MTP-4 joints, males had significantly greater joint space sizes than females for no traction (*p* = 0.039), manual traction (*p* = 0.002), and traction of 5 pounds (*p* = 0.004), 10 pounds, (*p* = 0.013), and 15 pounds (*p* = 0.024). There was no statistically significant difference in joint space size according to age or BMI for any MTP joints (*p* > 0.05). *Conclusions*: Among natural joint spaces without traction, the MTP-2 joint had the largest joint size while the MTP-5 joint had the smallest joint size. In MTP joint arthroscopy, a traction power of 10 lb is sufficient for appropriate distraction of all MTP joints. Less distraction power is required for males than for females, especially for the MTP-1 and MTP-4 joints.

## 1. Introduction

Metatarsophalangeal (MTP) joint arthroscopy has recently been used more broadly than ever before due to the development of high-definition small-joint arthroscopes and instruments. Diagnostic indications for MTP joint arthroscopy include swelling due to gout or rheumatoid arthritis, locking, and persistent joint pain. Therapeutic indications include arthrofibrosis, chondromalacia, degenerative joint disease, osteochondral lesions, osteophytes, a sesamoid pathology, synovitis, and osteochondrosis, such as Freiberg disease [1].

Since MTP joints have small sizes, appropriate distraction for metatarsophalangeal joint arthroscopy is paramount to increase visualization and decrease iatrogenic complications. Several different skeletal and soft tissue traction techniques have been introduced, including distraction methods using manual distraction [2], a mini external fixator [3], and Kirshner wire [4]. Other methods using a finger trap and positioning arm have also been devised [5].

This study aims to identify the appropriate distraction force for safe and effective MTP joint arthroscopy and to evaluate how patient factors such as gender, age, and BMI influence the required distraction force.

This study is also novel in that it utilizes a newly developed distraction device capable of real-time traction force monitoring, allowing precise and consistent distraction adjustment—a feature not available in previously reported techniques.

## 2. Materials and Methods

### 2.1. Materials

Sixty-seven patients (a total of 100 MTP joints, including 20 each of the different MTP joints) who underwent MTP joint arthroscopy from April 2013 to June 2020 were enrolled. This study was retrospectively conducted under our institution’s Institutional Review Board approval. The inclusion criterion was that patients underwent MTP joint arthroscopy due to rheumatoid arthritis (43 MTP joints in 20 patients), gouty arthritis (18 MTP joints in 16 patients), unknown arthritis (15 MTP joints in 12 patients), osteochondral lesions (19 MTP joints in 14 patients), pyogenic arthritis (4 MTP joints in 4 patients), or a loose body (1 MTP joint). The exclusion criterion was that patients underwent MTP joint arthroscopy due to joint contracture as a result of trauma, a burn, or diabetic foot joint infection. Autoclavable 1.9 mm, 30° high-definition arthroscopes (ConMed Linvatec, Largo, FL, USA) were used in this study. A small-joint shaver with 2.4 mm full-radius blades (Two-Button Ergo™ Shaver, ConMed Linvatec, Largo, FL, USA) was used. Instruments ranging in size from 1.6 to 2.8 mm in diameter included a probe, a basket, a grasper, and a curette. The minimum joint distance for MTP arthroscopy was defined at 2.8 mm, which was the sheath size of a 1.9 mm, 30° high-definition arthroscope. In order to avoid iatrogenic injury during surgery, the joint space should be larger than the outer diameter of the instrument. In this study, an MTP joint space of over 2.8 mm was defined as the possible size for MTP joint arthroscopy.

### 2.2. Methods

**Figure 1:**
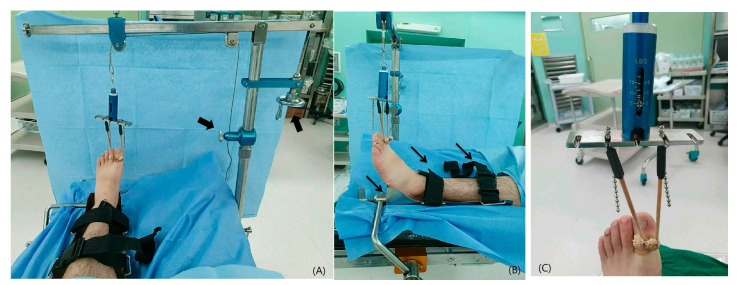
Pictures showing a novel traction machine. (**A**) There are two joints that can adjust the angle and traction power (black arrow). (**B**) For effective traction, the new device for fixing legs to the bed was developed and straps (narrow black arrows) were used to fix legs. (**C**) A device for measuring the strength of distraction in real time.

First, the patient was placed in a supine position and general spinal anesthesia was provided. A bump was created on the ipsilateral hip using a towel. Each toe was distracted using a string. The string was attached to the novel distraction machine originally developed by us. There are two joints for which it can adjust the angle. There is a device for measuring the strength of distraction. Therefore, the authors were able to check the strength of distraction in real time (Figure 1).

**Figure 2:**
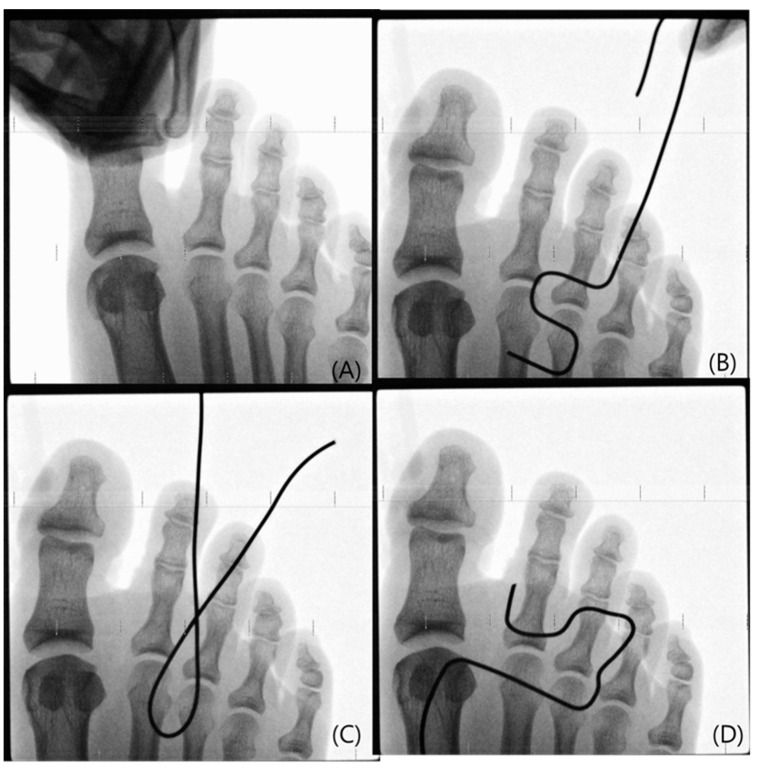
Anterior–posterior foot plain radiographs taken using a mini-fluoroscan with manual traction: (**A**), 5 lb (**B**), 10 lb (**C**), and 15 lb (**D**) of traction. These plain radiographs were computerized and used to measure the MTP joint space with a PACS system. The lines in these plain radiographs are wire lines representing the distraction pounds.

Anterior–posterior foot plain radiographs were taken using a mini-fluoroscan (InSight-FD, Hologic Inc., 35 Crosby Drive, Bedford, MA, USA) with different traction powers: no traction, manual traction, and traction of 5 pounds, 10 pounds, and 15 pounds. Plain radiographs were computerized and used to measure the MTP joint space with a Maro-view 5.4 Picture Archiving Communication System (PACS) (Health Tech Solutions, Inc., Tarpon Springs, FL, USA) (Figure 2). To amend the discrepancy between the real joint space and the joint space in a plain radiograph image, a foot plain radiograph was taken using a 1 cm Kirshner wire and then corrected by measuring the length in a PACS system. Age, gender, and body mass index were compared as associated factors. Age was divided into groups of under 30 years, 30~60 years, and over 60 years old. BMI was divided into under 23, 23~25, and over 25. To reduce measurement errors, measurements were taken twice and average values were calculated. We were able to achieve intra-observer and inter-observer reliabilities of 0.94 and 0.95, respectively.

### 2.3. Statistical Analysis

In statistical analysis, the Mann–Whitney test was used to compare gender. Additionally, the effect size (Cohen’s d) was calculated to determine the magnitude of differences, and 95% confidence intervals (CI) are provided for better interpretability. The Kruskal–Wallis test was used to compare age and body mass index.

For post hoc pairwise comparisons following the Kruskal–Wallis test, Bonferroni correction was applied to adjust for multiple comparisons, ensuring the control of type I error. The Bonferroni-adjusted significance level was set at *p* < 0.017 for comparisons among the three age groups (under 30, 30–60, over 60) and *p* < 0.017 for the three BMI groups (<23, 23–25, >25).

All statistical analyses were performed using SPSS ver. 17 (SPSS Inc., Chicago, IL, USA). Statistical significance was considered at *p* < 0.05 for single comparisons, and Bonferroni-adjusted *p*-values were used for multiple pairwise comparisons, with the results reported where applicable.

## 3. Results

### 3.1. Natural MTP Joint Space Size and Appropriate Distraction for a Safe MTP Joint Arthroscopy

Regarding natural MTP joint space sizes, the MTP-2 joint had the largest joint size (2.39 ± 0.37 mm). However, only 9 (45%) of 20 patients had sufficient joint sizes for us to perform MTP arthroscopy. The MTP-5 joint had the smallest joint size (1.59 ± 0.34 mm, Table 1).

Traction of 10 lb was an appropriate distraction force for MTP-1 joint (3.09 ± 0.03 mm) and MTP-4 joint (3.07 ± 0.47mm) arthroscopy. Traction of 5lb was an appropriate distraction force for MTP-2 (3.32 ± 0.60 mm), MTP-3 (2.89 ± 0.50 mm), and MTP-5 (2.97 ± 0.49 mm) joints. Manual traction was appropriate for the MTP-1 joint (2.89 ± 0.65 mm) and MTP-2 joint (3.13 ± 0.50 mm) based on the average joint space.

When the possible rate was defined as 70%, 10lb traction was an appropriate distraction in MTP-1 joint, MTP-3 joint, MTP-4 joint, and MTP-5 joint (possible rate 92.5%) arthroscopy. We found that 5 lb traction and manual traction were only appropriate for the MTP-2 joint (Table 1).

### 3.2. Mean MTP Joint Space Size and Distraction According to Gender Difference

For the MTP-1 and MTP-4 joints, males had significantly greater joint space sizes than females for no traction, manual traction, and tractions of 5 lb, 10 lb, and 15 lb (Table 2). Effect sizes (Cohen’s d) indicated moderate to large differences, ranging from d = 0.6 to 1.2. The 95% confidence intervals (CIs) for these differences were also calculated and are presented in Table 3. In the MTP-3 joint, males had significantly greater in the space sizes than females for no traction (*p* = 0.049) and traction of 5 lb (*p* = 0.003). There was no significant gender difference in MTP-2 or MTP-5 joint space size (Table 2).

### 3.3. Mean MTP Joint Space Size and Distraction According to Age and BMI Difference

There was no statistically significant difference in joint space size according to age or BMI for any MTP joints (Table 2).

**Table 3:** Effect size estimation (e.g., Cohen’s d) and confidence intervals for gender differences in the 1st MTP joint.

MTP-1 Joint					
Gender					
	Male (11)	Female (9)	*p*-Value	Cohen’s d	95% CI
No traction	2.14 ± 0.40	1.88 ± 0.28	**0.039**	0.73	(–0.06, 0.58)
Manual traction	3.16 ± 0.68	2.53 ± 0.40	**0.002**	1.10	(0.11, 1.1)
5 lb traction	2.99 ± 0.78	2.36 ±0.26	**0.004**	1.03	(0.10, 1.15)
10 lb traction	3.31 ± 0.75	2.78 ± 0.22	**0.013**	0.91	(0.03, 1.03)
15 lb traction	3.64 ± 0.76	3.16 ± 0.28	**0.024**	0.80	(–0.03, 1.00)

## 4. Discussion

Arthroscopic examination of the great toe (1st toe) MTP joint was first described by Watanabe [6]. The first case series was reported by Ferkel and Van Buechen in 1991 [7]. MTP joint arthroscopy has recently been used more broadly than ever before due to the development of high-definition small-joint arthroscopes and instruments. Its indications include swelling due to gout or rheumatoid arthritis, arthrofibrosis, chondromalacia, degenerative joint disease, osteochondral lesions, osteophytes, a sesamoid pathology, and synovitis [1].

Due to the small size of the MTP joint, appropriate distraction is critical to ensuring adequate visualization and minimizing iatrogenic injury during arthroscopy. Davies et al. [2] have reported that the hallux can be suspended using a large Chinese finger trap with traction of approximately 2.7 kg over a pulley attached to the opposite side of the operating table. However, toes are shorter than fingers, making it difficult for a finger trap to effectively perform traction. Derner et al. [3] have reported that traction can be applied to the great toe (1st toe) either with an assistant or a mini external fixator if necessary. Siclari [4] et al. have also reported that the hallux can be suspended using a Kirschner wire in the second phalange from an articulated arm on the operating table. However, a mini external fixator and a Kirschner wire have the disadvantage of being invasive. Hull et al. [8] have reported that an axial skin traction of 22 N (5 lb) can be applied via skin traction through the hallux proximal phalanx/interphalangeal joint. Hunt et al. [9] have also reported that a 4 × 8 gauze can be used in a finger trap fashion for manual distraction. However, manual traction has the disadvantage that the traction strength of an assistant is inaccurate and inconsistent.

Compared to Davies et al. [2], who recommended approximately 2.7 kg (about 6 lb) of traction using a Chinese finger trap system, this study found that 5 lb was sufficient for MTP-2, MTP-3, and MTP-5 joints, while 10 lb was required for MTP-1 and MTP-4 joints. This discrepancy may stem from differences in traction techniques. The Chinese finger trap method applies purely axial traction, whereas the novel traction device used in this study allows for real-time monitoring and adjustment of both axial and angular forces, ensuring consistent and controlled distraction throughout the procedure. This ability to fine-tune distraction may reduce the overall force required to achieve a sufficient joint space. In addition, the difference in required distraction force between males and females observed in this study has not been well-described in the previous literature. Males demonstrated larger baseline joint spaces and required lower traction forces to achieve sufficient arthroscopic visualization, particularly in the MTP-1, MTP-3, and MTP-4 joints. This finding highlights the importance of tailoring traction forces to patient-specific factors, which has been underappreciated in prior studies.

The findings of this study have direct implications for optimizing arthroscopic techniques in various clinical scenarios. For example, in patients with gouty arthritis or rheumatoid arthritis, where synovial hypertrophy and joint effusion frequently narrow the joint space, applying excessive traction to overcome these obstacles may increase the risk of traction-related nerve injury or chondral damage. By establishing patient- and joint-specific traction guidelines, this study provides a practical framework for achieving optimal visualization while minimizing risks.

Traction of five pounds is generally known for an appropriate traction power. However, this study showed that traction of 5 pounds is appropriate only for MTP-2, -3, and -5 joints. Meanwhile, traction of 10 pounds is thought to be an effective traction power for all MTP joints based on the average joint space.

Few studies have been conducted on the size of the MTP joint. The joint space without traction measured in this study is meaningful as it is the first study on real patients. In this study, although the MTP-2 joint space without traction was the largest joint, it was smaller than the standard 2.8 mm. It provides strong scientific evidence for why appropriate traction is needed for MTP joint arthroscopy.

MTP-1, MTP-3, and MTP-4 joints of males had statistically significantly greater joint space sizes than those of females. However, there was no statistically significant difference in joint space size according to age or BMI for any MTP joints. Therefore, in the case of men, regardless of age and BMI, it is possible to perform traction with less strength. In particular, in the case of men, because the size of the joint without traction was large, enough joint space could be secured with just 5 pounds of traction. Mass et al. [10] have reported plantar plate anatomy related to the MTP joint stability of lesser toes, focusing the importance of the plantar plate anatomy and integrity for MTP joint stability. The joint space in traction depends on the strength and width of the plantar plate. Generally, males have thicker ligament tissues (including the plantar plate) than females. Thus, males are expected to require more traction power than females. However, sufficient joint space was secured with less distraction power for males, especially for the MTP-1 joint. The authors believe that joint space can be secured with less force in males because their sizes of joints are larger than those of females without traction.

In general, it is expected that younger people will need more traction power because they have stronger ligament structures, including the plantar plate. In this study, there was no significant difference in traction power needed according to age for any MTP joints. There is some possibility that age can have an influence during small-joint arthroscopy. More studies are needed on the relationship between age and traction power. One of our hypotheses is that the higher the weight or BMI of a patient, the more traction power is needed. However, the results showed no significant correlation between weight and traction power. Few studies have reported the correlation between weight and the elasticity of ligaments. Thus, more studies are needed in the future.

This study has some limitations. First, a small number of patients were enrolled. Second, although real-time traction forces were recorded, this study did not directly assess the biomechanical effects of these forces on the plantar plate, collateral ligaments, or articular cartilage. Future studies using cadaveric models or in vivo pressure sensors could help elucidate how varying distraction forces influence intra-articular pressure and ligament strain. Third, age and BMI were analyzed as categorical variables, which may have limited the detection of more nuanced relationships between these factors and traction requirements.

Future research should include prospective trials to validate these findings across a broader patient population with varying pathologies, including inflammatory arthritis, degenerative arthritis, and post-traumatic arthritis. Additionally, biomechanical modeling, such as finite element analysis, could provide valuable insights into how traction forces are distributed across the plantar plate and adjacent structures. Cadaveric studies could also be employed to directly visualize the effects of different distraction forces on soft tissue integrity and joint congruity. Furthermore, investigating the impact of disease severity (e.g., degree of synovitis or cartilage degeneration) on the optimal traction force could support efforts to further refine the clinical guidelines for MTP joint arthroscopy.

## 5. Conclusions

For natural joint spaces without traction, the MTP-2 joint had the largest joint size while the MTP-5 joint had the smallest joint size. In MTP joint arthroscopy, a traction power of 10 lb is sufficient for appropriate distraction for all MTP joints, while a traction power of 5 lb is an appropriate power for distraction of the MTP-2 joint. Less distraction power is required for males than for females, especially for MTP-1, MTP-3, and MTP-4 joints.

## Figures and Tables

**Table 1:** Joint space and possible rate with traction.

	MTP-1 Joint Space (mm)	MTP-2 Joint Space (mm)	MTP-3 Joint Space (mm)	MTP-4 Joint Space (mm)	MTP-5 Joint Space (mm)
No traction	2.03 ± 0.37	2.39 ± 0.37	2.01 ± 0.47	1.76 ± 0.33	1.59 ± 0.34
Manual traction	2.89 ± 0.65	3.13 ± 0.50	2.70 ± 0.48	2.49 ± 0.48	2.43 ± 0.51
5 lb traction	2.73 ± 0.68	3.32 ± 0.60	2.89 ± 0.50	2.67 ± 0.44	2.97 ± 0.49
10 lb traction	3.09 ± 0.03	3.76 ± 0.72	3.56 ± 0.58	3.07 ± 0.47	3.52 ± 0.47
15 lb traction	3.43 ± 0.63	4.07 ± 0.79	3.73 ± 0.56	3.64 ± 0.64	4.04 ± 0.62

**Table 2:** MTP joint size according to distraction under associated factors.

	MTP-1 Joint										
	Gender			Age				BMI			
	Male (11)	Female (9)	*p*-value	<30 (6)	30~60 (7)	>60 (7)	*p*-value	<23 (6)	23~25 (5)	>25 (9)	*p*-value
No traction	2.14 ± 0.40	1.88 ± 0.28	**0.039**	1.92 ± 0.44	1.99 ± 0.32	2.18 ± 0.37	0.196	1.89 ± 0.39	2.09 ± 0.32	2.09 ± 0.40	0.314
Manual traction	3.16 ± 0.68	2.53 ± 0.40	**0.002**	2.84 ± 0.523	2.91 ± 0.70	2.92 ± 0.76	0.956	2.95 ± 0.62	2.74 ± 0.49	2.93 ± 0.75	0.737
5 lb traction	2.99 ± 0.78	2.36 ± 0.26	**0.004**	2.59 ± 0.56	2.70 ± 0.72	2.87 ± 0.79	0.891	2.52 ± 0.49	2.70 ± 0.58	2.86 ± 0.83	0.759
10 lb traction	3.31 ± 0.75	2.78 ± 0.22	**0.013**	3.00 ± 0.42	3.09 ± 0.66	3.16 ± 0.79	0.714	2.94 ± 0.39	3.15 ± 0.43	3.15 ± 0.82	0.312
15 lb traction	3.64 ± 0.76	3.16 ± 0.28	**0.024**	3.22 ± 0.54	3.51 ± 0.60	3.54 ± 0.77	0.140	3.28 ± 0.45	3.61 ± 0.48	3.45 ± 0.80	0.279
	**MTP-2 Joint**										
	Gender			Age				BMI			
	Male (11)	Female (9)	*p*-value	<30 (6)	30~60 (7)	>60 (7)	*p*-value	<23 (6)	23~25 (5)	>25 (9)	*p*-value
No traction	2.45 ± 0.40	2.30 ± 0.31	0.218	2.32± 0.34	2.35 ± 0.35	2.49 ± 0.45	0.322	2.34 ± 0.29	2.34 ± 0.32	2.43 ± 0.44	0.749
Manual traction	3.21 ± 0.58	3.28 ± 0.37	0.257	3.28 ± 0.37	2.90 ± 0.50	3.26 ± 0.55	0.097	3.10 ± 0.43	3.16 ± 0.65	3.15 ± 0.49	0.954
5 lb traction	3.46 ± 0.65	3.12 ± 0.49	0.074	3.22 ± 0.65	3.25 ± 0.55	3.49 ± 0.63	0.408	3.07 ± 0.48	3.60 ± 0.62	3.34 ± 0.63	0.102
10 lb traction	3.91 ± 0.80	3.56 ± 0.58	0.136	3.80 ± 0.85	3.72 ± 0.52	3.78 ± 0.86	0.990	3.58 ± 0.56	4.11 ± 0.73	3.72 ± 0.80	0.221
15 lb traction	4.20± 0.88	3.90 ± 0.66	0.242	4.12 ± 0.97	4.02 ± 0.58	4.08 ± 0.90	0.980	3.91 ± 0.70	4.46 ± 0.79	3.99 ± 0.84	0.208
	**MTP-3 joint**										
	Gender			Age				BMI			
	Male (11)	Female (9)	*p*-value	<30 (6)	30~60 (7)	>60 (7)	*p*-value	<23 (6)	23~25 (5)	>25 (9)	*p*-value
No traction	2.14 ± 0.53	1.82 ± 0.33	**0.028**	1.96 ± 0.38	1.96 ± 0.46	2.10 ± 0.57	0.740	1.88 ± 0.41	2.02 ± 0.29	2.07 ± 0.57	0.553
Manual traction	2.79 ± 0.50	2.56 ± 0.45	0.146	2.72 ± 0.47	2.65 ± 0.50	2.72 ± 0.50	0.918	2.72 ± 0.49	2.58 ± 0.50	2.73 ± 0.50	0.723
5 lb traction	3.08 ± 0.52	2.62 ± 0.32	**0.008**	2.73 ± 0.50	2.89 ± 0.41	3.01 ± 0.58	0.383	2.72 ± 0.37	2.99 ± 0.50	2.93 ± 0.57	0.416
10 lb traction	3.57 ± 0.57	3.31 ± 0.58	0.156	3.43 ± 0.63	3.45 ± 0.55	3.50 ± 0.62	0.957	3.41 ± 0.51	3.55 ± 0.48	3.45 ± 0.69	0.924
15 lb traction	3.86 ± 0.58	3.55 ± 0.52	0.096	3.74 ± 0.63	3.67 ± 0.46	3.79 ± 0.64	0.941	3.67 ± 0.52	3.98 ± 0.52	3.65 ± 0.60	0.202
	**MTP-4 join**										
	Gender			Age				BMI			
	Male (11)	Female (9)	*p*-value	<30 (6)	30~60 (7)	>60 (7)	*p*-value	<23 (6)	23~25 (5)	>25 (9)	*p*-value
No traction	1.84 ± 0.32	1.65 ± 0.31	0.063	1.80 ± 0.30	1.65 ± 0.34	1.85 ± 0.35	0.307	1.72 ± 0.32	1.65 ± 0.30	1.84 ± 0.34	0.405
Manual traction	2.64 ± 0.53	2.27 ± 0.34	**0.015**	2.50 ± 0.44	2.43 ± 0.56	2.54 ± 0.47	0.534	2.45 ± 0.50	2.34 ± 0.57	2.58 ± 0.49	0.361
5 lb traction	2.82 ± 0.46	2.48 ± 0.32	**0.014**	2.57 ± 0.45	5.65 ± 0.39	2.80 ± 0.49	0.391	2.59 ± 0.28	2.79 ± 0.56	2.67 ± 0.47	0.617
10 lb traction	3.23 ± 0.50	2.85 ± 0.32	**0.010**	3.05 ± 0.59	3.02 ± 0.31	3.15 ± 0.56	0.774	3.01 ± 0.40	3.25 ± 0.49	3.03 ± 0.50	0.429
15 lb traction	3.80 ± 0.69	3.36 ± 0.48	**0.018**	3.51 ± 0.73	3.67 ± 0.44	3.74 ± 0.80	0.669	3.57 ± 0.51	3.87 ± 0.10	3.58 ± 0.71	0.500
	**MTP-5 joint**										
	Gender			Age				BMI			
	Male (11)	Female (9)	*p*-value	<30 (6)	30~60 (7)	>60 (7)	*p*-value	<23 (6)	23~25 (5)	>25 (9)	*p*-value
No traction	1.71 ± 0.33	1.44 ± 0.28	0.110	1.60 ± 0.36	1.54 ± 0.33	1.66 ± 0.38	0.668	1.56 ± 0.36	1.58 ± 0.36	1.62 ± 0.33	0.893
Manual traction	2.44 ± 0.49	2.41 ± 0.56	0.857	2.34 ± 0.48	2.54 ± 0.60	2.40 ± 0.44	0.335	2.53 ± 0.53	2.33 ± 0.55	2.42 ± 0.50	0.680
5 lb traction	3.00 ± 0.50	2.94 ± 0.50	0.723	2.91 ± 0.46	3.06 ± 0.61	2.93 ± 0.40	0.686	3.17 ± 0.41	2.87 ± 0.58	2.90 ± 0.49	0.249
10 lb traction	3.59 ± 0.48	3.41 ± 0.47	0.252	3.42 ± 0.53	3.60 ± 0.47	3.50 ± 0.46	0.624	3.63 ± 0.44	3.54 ± 0.43	3.43 ± 0.53	0.534
15 lb traction	4.18 ± 0.66	3.85 ± 0.53	0.102	3.88 ± 0.60	4.12 ± 0.60	4.09 ± 0.70	0.431	4.12 ± 0.45	4.08 ± 0.58	3.97 ± 0.75	0.782

(number) means case number.

## Data Availability

The data presented in this study are available upon request from the corresponding author.
